# Trends in research on acute lung injury/acute respiratory distress syndrome associated with viral pneumonia from 1992 to 2022: a 31-year bibliometric analysis

**DOI:** 10.3389/fmed.2023.1158519

**Published:** 2023-06-09

**Authors:** Luofei Zhang, Shenghui Mei, Bin Zhu, Zhigang Zhao

**Affiliations:** ^1^Department of Pharmacy, Beijing Tiantan Hospital, Capital Medical University, Beijing, China; ^2^Department of Clinical Pharmacology, College of Pharmaceutical Sciences, Capital Medical University, Beijing, China

**Keywords:** acute lung injury, acute respiratory distress syndrome, bibliometrics, viral pneumonia, virus

## Abstract

**Purpose:**

Acute lung injury/acute respiratory distress syndrome (ALI/ARDS) is a dangerous symptom in patients with severe viral pneumonia. This study aims to comprehensively review the cooperation and influence of countries, institutions, authors and co-cited journals/authors/references and keywords in the field of ALI/ARDS associated with viral pneumonia from the perspective of bibliometrics, evaluate the clustering evolution of knowledge structure, and find hot trends and emerging topics.

**Methods:**

Publications on ALI/ARDS associated with viral pneumonia published from January 1, 1992 to December 31, 2022 were extracted from the Web of Science core collection. The document type was limited to original article or review, with the language set to English. Citespace was used to conduct the bibliometric analysis.

**Results:**

A total of 929 articles were included, and the number of them generally increased over time. The countries with the most published articles in this field are the United States (320 papers) and Fudan University is the institution (15 papers) with the most research results. The *New England Journal of Medicine* was the most frequently co-cited journal, while the most influential co-cited journal was *American Journal of Clinical Pathology*. Reinout A Bem and Cao Bin were the most prolific author, but there was no leader in this field. The keywords with both high frequency and high centrality were “pneumonia” (Freq = 169, Central = 0.15), “infection” (Freq = 133, Central = 0.15), “acute lung injury” (Freq = 112, Central = 0.18), “respiratory distress syndrome” (Freq = 108, Central = 0.24), and “disease” (Freq = 61, Central = 0.17). “Failure” was the first keyword with citation bursts. Meanwhile, “coronavirus,” “cytokine storm” and “respiratory syndrome coronavirus” continue to burst.

**Conclusion:**

Although there was a surge in literature since 2020, attentions to ALI/ARDS associated with viral pneumonia were still insufficient over last three decades. The communication and cooperation among countries, institutions and authors need to be further strengthened.

## Introduction

Acute lung injury (ALI)/acute respiratory distress syndrome (ARDS) is a critical clinical syndrome with high morbidity and mortality (1). In addition to inhalation lung injury, pulmonary contusion, sepsis and other causes, viral pneumonia is one of the complex causes of ALI/ARDS (2). The treatment and management of ALI/ARDS may be different due to various causes. Meanwhile, the outbreak of coronavirus epidemic in 2019 has raised more attention to ALI/ARDS associated with viral pneumonia.

To humans, the most common type of infection is respiratory infection, in which viral infections dominate (3). Influenza virus, rhinovirus, and adenovirus frequently cause viral pneumonia, which is an important cause of morbidity and mortality (4). The emergence of severe acute respiratory syndrome (SARS), avian influenza A (H5N1) virus, and pandemic influenza A (H1N1) virus in 2009 re-emphasized the important role of respiratory viruses as a cause of severe pneumonia (5). Respiratory syncytial virus, rhinovirus, human metapneumovirus, human bocavirus and parainfluenza virus are the common pathogens causing viral pneumonia in children, while influenza virus, rhinovirus and coronavirus are the main pathogens causing viral pneumonia in adults (5). As well-known that RNA viruses and coronaviruses (CoVs) were considered to be two of the relatively harmless respiratory pathogens in the past (6); but in the last few decades, CoVs have been associated with 3 new severe diseases, namely SARS (7), Middle East respiratory syndrome coronavirus (8) (MERS-CoV) and ongoing coronavirus infectious disease 19 (9) (COVID-19) outbreaks. The clinical manifestations of the viral pneumonia range from mild to severe pneumonia, and severe cases could cause ALI/ARDS. ALI/ARDS associated with viral pneumonia may have both the characteristics of high infectivity of viral pneumonia and high mortality of ALI/ARDS, which will have a serious impact on human public health worldwide. Patients with ALI/ARDS associated with viral pneumonia need more attention.

Bibliometrics is a subject of quantitative analysis, which can help researchers identify the current status and frontiers in a particular topic or research field, providing new insights and directions for future research (10). Citespace is a commonly used bibliometric visualization tool for data analysis and visualization, which can promote readers’ subjective understanding (11). Although bibliometric analysis of viral pneumonia (6) or ALI/ARDS (12) have been performed, no bibliometric study of ALI/ARDS associated with viral pneumonia has been performed. This study conducted a bibliometric analysis of publications, including countries/regions, institutions, authors, co-cited journals/authors/references and keywords, so as to explore the research status, hotspots and frontiers, and draw knowledge maps using Citespace to serve as a reference for relevant future research.

## Methods

### Data acquisition

All literatures were retrieved from the Web of Science (WOS). Literature probe was conducted in the Science Citation Index Expanded (SCI-Expanded) of the Web of Science Core Collection (WOSCC) on 6 January 2023, employing the following terms in the topic: (“viral pneumonia*”) AND (“acute lung injury*” OR “ALI” OR “acute respiratory distress syndrome*” OR “adult respiratory distress syndrome*” OR “respiratory distress syndrome*” OR “shock lung” OR “human ARDS” OR “ARDS”). Original articles or reviews in English from January 1, 1992 to December 31, 2022 were finally included, while conference abstracts, proceedings papers, book chapters, news, presentations and so on were excluded. Ultimately, a total of 929 records were acquired. Full records and cited references were downloaded in plain text format.

### Data analysis

Bibliometric analysis included co-authorship and co-citation analysis. Co-authorship analysis refers to the evaluation of the relationship among items based on the number of coauthored documents, which were considered one of the most tangible indicators to evaluate collaboration trends and identify leading countries, institutions, and authors (13). Co-citation refers to when two or more references/authors/journals are simultaneously cited in one or more subsequent papers, which is called a co-cited relationship between them (13). Co-cited count is defined as the frequency with which two or more references/authors/journals are cited together by subsequently published articles. The bibliometrics online analysis platform^1^ was applied to analyze the publication trend and national cooperation network of the included literatures in diverse countries. Citespace is an optimal collaborative network analysis tool for connecting the various publication features created by Chaomei Chen, and Citespace 6.1.R6, 64bit (Drexel University, Philadelphia, Pennsylvania, United States) software was used for visual analysis in this study, encompassing countries/regions, institutions, authors, co-cited journals/authors/references and keywords. Parameter settings of Citespace: time span (1992–2022), years per slice (1), look back years (LBY = 5), and selection criteria (g-index: *k* = 25 for country, institution, author, keyword, and co-cited journal; g-index: *k* = 15 for co-cited author and reference).

## Results

### Distribution of articles by publication years/countries

**Figure 1:**
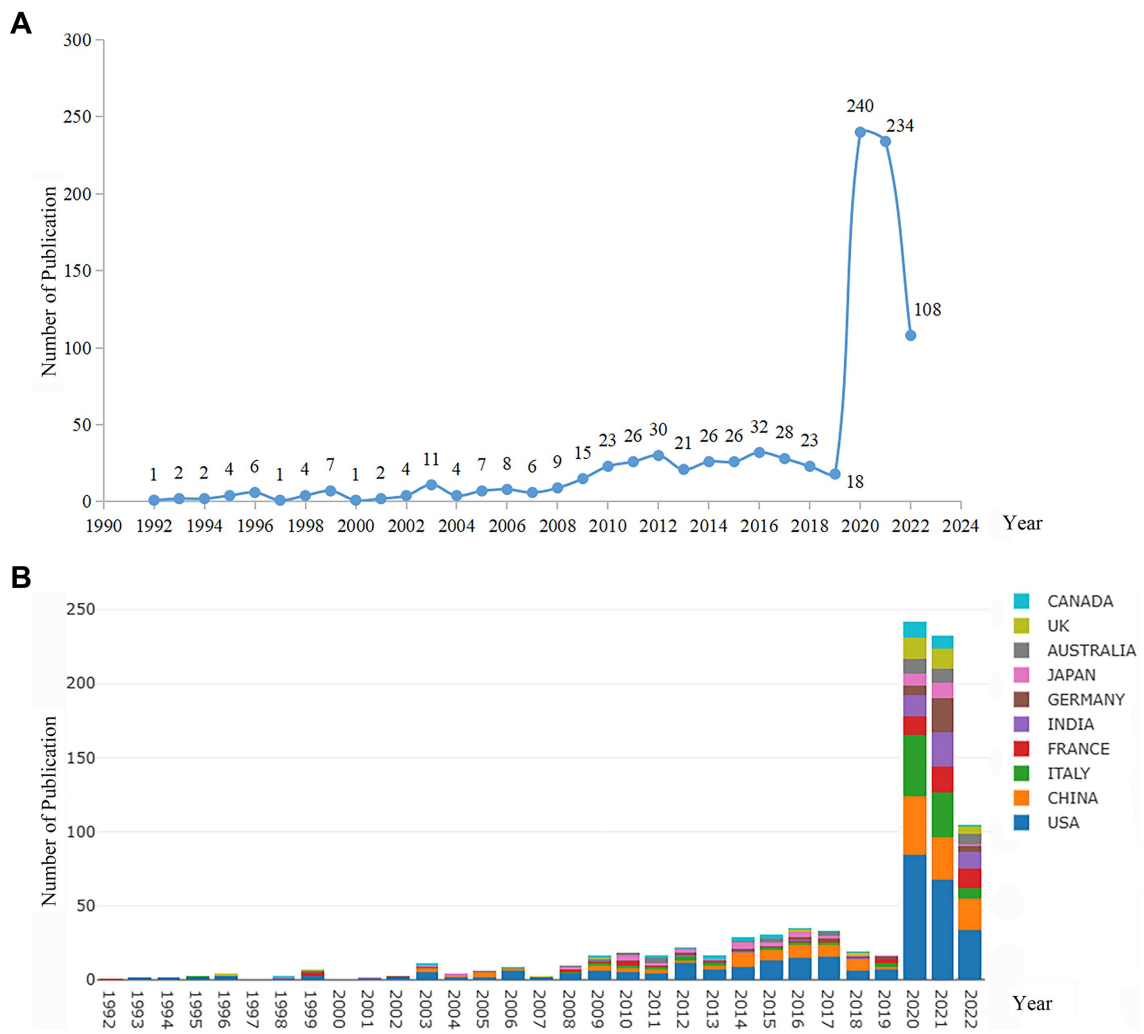
**(A)** Distribution of annual published literature related to ALI/ARDS associated with viral pneumonia from 1992 to 2022; **(B)** Annual publication volume and growth trend of ALI/ARDS associated with viral pneumonia in the top 10 countries from 1992 to 2022.

A total of 929 publications were included for additional analysis, including 621 original articles and 308 reviews. As shown in Figure 1A, the publications on ALI/ARDS associated with viral pneumonia can be roughly divided into three stages: stage I (1992–2008), stage II (2009–2019), and stage III (2020–2022). In the first stage, the progress was extremely slow, which lasted for 17 years, with an average annual publication number of 4.6. The second stage (time span of 11 years) began to increase gradually, with an average annual publication number of 24.4, and the highest number of annual publications was 32 in 2016. The third stage showed explosive growth, and the annual publications number reached 240 in 2020. The total number of stage III publications was 584, accounting for 62.9% of all included publications, but the duration was no more than 3 years, accounting for 10.0% of the total time span. The bar chart (Figure 1B) showed the top 10 countries by volume of publications in ALI/ARDS associated with viral pneumonia research from 1992 to 2022. The first paper related to this field was published in France in 1992. The United States was identified as the pioneer in this field. China was active in all stages. Italy contributed little to the number of papers published in this field in the first two stages, but it was rapid in the third stage.

### Distribution of countries/regions and institutions

**Figure 2:**
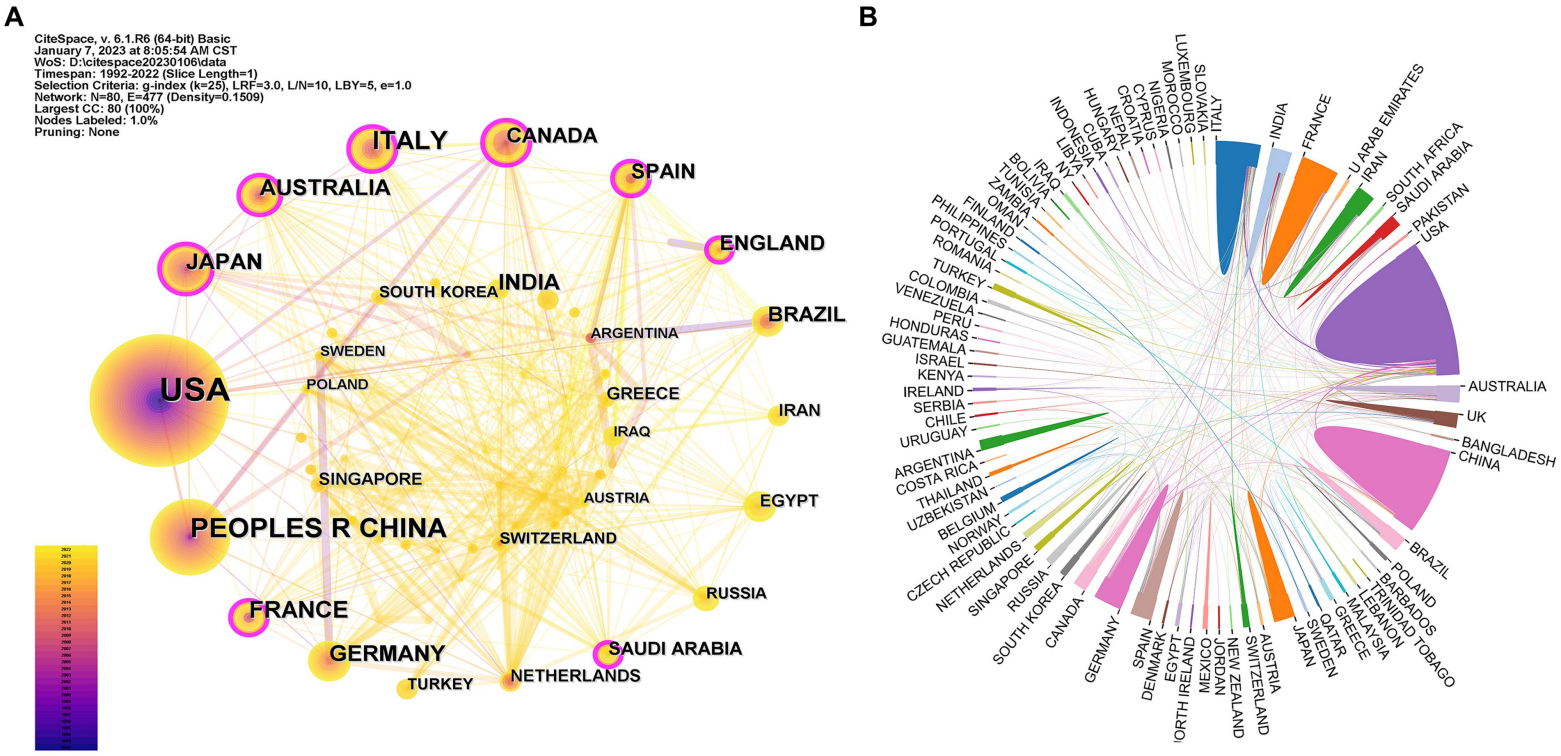
Collaborative network of countries/regions. **(A)** Collaborative network of countries/regions by Citespace. Each node represents a country/region. The bigger the node, the more articles the country/region published. The thicker the connection line, the closer the relationship. The cooler colors indicate more distant time, while warmer colors indicate more recent time. A node with purple ring means high betweenness centrality (>0.1); **(B)** Collaborative network of countries/regions by a bibliometrics online analysis platform (http://bibliometric.com/). Each area represents a country/region. The larger the area, the more articles the country/region published. Lines indicate connections between countries/regions.

National/regional cooperation network in research on ALI/ARDS associated with viral pneumonia are shown in Figure 2. The top 15 countries/regions in terms of number of publications are shown in Table 1. The United States ranked first with 320 papers, followed by China with 158 publications, and the number of papers published by other countries/regions did not exceed 100. A purple ring at the edge of the node indicates that the node has a high degree of betweenness centrality (>0.1), which means that it plays a key role in connecting the connected nodes. Purple rings could be observed at eight nodes in Figure 2A, which represented these countries/regions ranked in descending order of betweenness centrality: Canada (0.15), Australia (0.14), England (0.14), Spain (0.14), France (0.13), Italy (0.12), Saudi Arabia (0.12), and Japan (0.11).

**Table 1:** The top 15 countries/regions and institutions contributed to publications on ALI/ARDS associated with viral pneumonia research.

Rank	Country	Output [*n* (%)]	Institution	Output [*n* (%)]
1	USA	320 (34.44)	Fudan University	15 (1.61)
2	China	158 (17.01)	Northwestern University	13 (1.40)
3	Italy	95 (10.23)	Capital Medical University	11 (1.18)
4	France	56 (6.03)	Harvard Medical School	11 (1.18)
5	India	51 (5.49)	University Pittsburgh	11 (1.18)
6	Germany	50 (5.38)	University Toronto	10 (1.08)
7	Japan	47 (5.06)	Johns Hopkins University	10 (1.08)
8	Australia	45 (4.84)	Monash University	9 (0.97)
9	Canada	42 (4.52)	National and Kapodistrian University of Athens	9 (0.97)
10	Engand	38 (4.09)	Stanford University	9 (0.97)
11	Brazil	35 (3.77)	University Washington	8 (0.86)
12	Spain	34 (3.66)	University Hong Kong	7 (0.75)
13	Saudi Arabia	22 (2.37)	Chinese Academy Medical Science	7 (0.75)
14	Netherlands	20 (2.15)	University Tehran Medical Science	7 (0.75)
15	Iran	19 (2.05)	All India Institute Medical Science	6 (0.65)

Moreover, Table 1 also exhibits the top 15 most productive institutions that contributed to the research on ALI/ARDS associated with viral pneumonia. Research institutions with a high frequency of publications are identified as influential institutions (14). All of them were universities and came from seven countries/regions: the United States (6), China (4), Canada (1), Australia (1), Greece (1), Iran (1), and India (1). Universities have played an important role in this field. Fudan University (China) ranked the first with 15 publications, followed by Norhtwestern University (the United States) with 13 publications, and three universities [Capital Medical University (China), Harvard Medical School (the United States), University Pittsburgh (United States)] had 11 publications, also indicating that contributions from the United States and China cannot be ignored in this field. However, none of the institutions in this field had a betweenness centrality greater than 0.1, indicating that the cooperation among institutions needs to be strengthened. The overall picture of the institutional network is presented in Supplementary Figure S1A, while the largest institutional cooperation network is shown in Supplementary Figure S1B, which further confirms the fact that the degree of inter-agency cooperation in this field needs to be strengthened.

### Distribution by co-cited journals, authors, and co-cited authors

**Figure 3:**
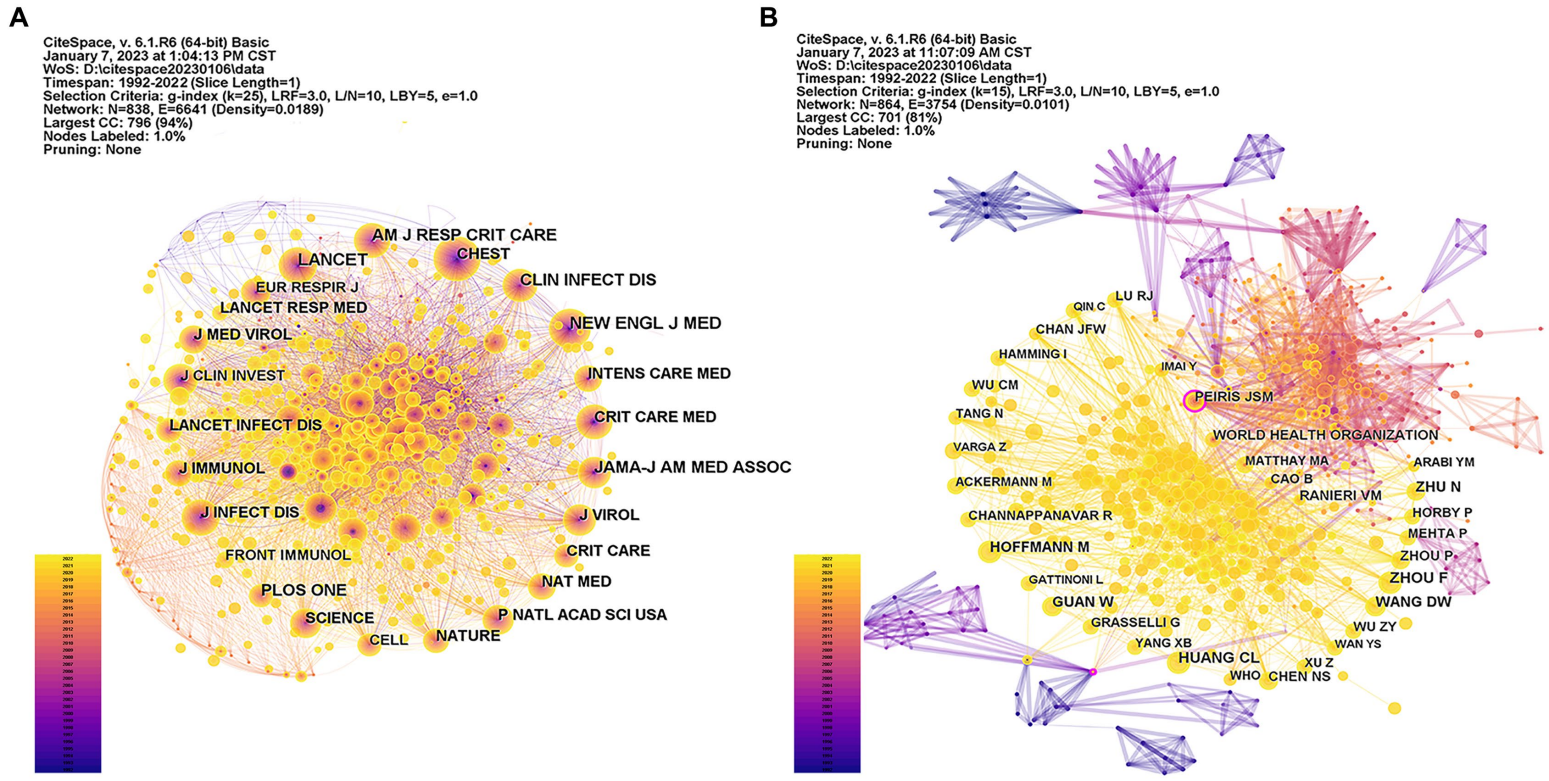
**(A)** Network of co-cited journals; **(B)** Network of co-cited authors. Each node represents a co-cited journal/author. The larger the node, the more frequently it is co-cited. A node with purple ring means high betweenness centrality (>0.1). The thicker the purple circle, the stronger the betweenness centrality.

Eight hundred and thirty-eight co-cited journals (nodes) and 6,641 collaborative relationships (connections) are included in Figure 3A. Co-cited journals are those cited together by other researchers. Through co-citation of journal analysis, the distribution of key knowledge sources can be obtained in a field (15). The top 15 co-cited journals with the highest frequency of ALI/ARDS associated with viral pneumonia research are presented in Table 2. The *New England Journal of Medicine* (631), *Lancet* (568), and *JAMA-Journal of the American Medical Association* (473) were the three most frequently co-cited journals, but none of the co-cited journals had a betweenness centrality greater than 0.1, indicating that the influence of co-cited journals needs to be improved in this field.

**Table 2:** The top 15 active co-cited journals, authors, and co-cited authors in the field of ALI/ARDS associated with viral pneumonia research.

Rank	Co-cited Journal^a^	Count	Centrality	Rank	Authors	Count	Centrality	Rank	Co-cited authors	Count	Centrality
1	*New Engl J Med*	631	0.06	1	Bem RA	5	0.00	1	Huang CL	147	0.01
2	*Lancet*	568	0.02	2	Cao B	5	0.00	2	Zhou F	141	0.03
3	*Jama-J Am Med Assoc*	473	0.02	3	Chen DF	4	0.00	3	Guan W	109	0.01
4	*Am J Resp Crit Care*	405	0.02	4	Batiha GES	4	0.00	4	Wang DW	108	0.03
5	*Plos One*	372	0.00	5	Zhu HY	4	0.00	5	Hoffmann M	92	0.03
6	*Clin Infect Dis*	360	0.03	6	Zhang L	3	0.00	6	Zhu N	91	0.00
7	*Nature*	338	0.04	7	Bime C	3	0.00	7	Ranieri VM	89	0.04
8	*Chest*	309	0.03	8	Liu L	3	0.00	8	Zhou P	89	0.04
9	*J Virol*	294	0.04	9	Shi XL	3	0.00	9	Peiris JSM	77	**0.11**
10	*J Infect Dis*	292	0.02	10	Luyt CE	3	0.00	10	Chen NS	73	0.03
11	*Intens Care Med*	283	0.01	11	Akkanti B	3	0.00	11	World health organization	72	0.06
12	*Science*	280	0.02	12	Moni MA	3	0.00	12	Wu ZY	67	0.01
13	*P Natl Acad Sci Usa*	273	0.03	13	Al-kuraishy HM	3	0.00	13	Cao B	65	0.05
14	*Lancet Resp Med*	265	0.00	14	Rodriguez-morales AJ	2	0.00	14	Channappanavar R	64	0.01
15	*Nat Med*	260	0.03	15	Wang T	2	0.00	15	Wu CM	62	0.00

a Journal names according to Index of Medical Journal Abbreviations. Bold for high centrality (>0.1).

The top 15 active authors and co-cited authors in the field of ALI/ARDS associated with viral pneumonia research are also shown in Table 2. Bem RA was the most active author in the field with 5 published papers, followed by Cao B with 5 papers and Chen DF with 4 papers. Most authors published 1 or 2 papers, indicating that only a few authors made a sustained contribution to this field. The overall picture of the author network is presented in Supplementary Figure S2A, Supplementary Figure S2B shows the largest author cooperation network. The network map of authors involved in ALI/ARDS associated with viral pneumonia research showed a low map density (Density = 0.0049) (Supplementary Figure S2A), suggesting that research groups were relatively dispersed, with scattered authors and that mutual cooperation needed strengthening to share the research results of ALI/ARDS associated with viral pneumonia in different regions and disciplines, and promote the continuous progress of research (15). In addition, due to genetic diversity, inconsistencies in population composition, differences in virus strains, and potential inconsistencies in treatment efficacy or prognosis in different regions, collaboration between different research teams is very important for the comprehensive study of ALI/ARDS associated with viral pneumonia. The betweenness centrality of all authors was 0.00, indicating that most of the authors’ influence was still at a low level and the degree of cooperation between authors was not enough.

Compared with the author collaboration network (Density = 0.0049), the density of the co-cited author network (Density = 0.0101) was significantly increased (Figure 3B). Huang CL ranked the highest about co-cited counts (147), followed by Zhou *F* (141), Guan W (109), and Wang DW (108), while the remaining co-cited authors were co-cited less than 100 times (Table 2). There are two nodes with the purple circle shown in Figure 3B: The nodes with the purple circle in the upper right of Figure 3B represents the co-cited author Peiris JSM, with a high centrality of 0.11; The node with the purple circle in the lower left of Figure 3B represents the co-cited author, named Ashbaugh DG, who had high betweenness centrality (0.16) despite being co-cited by only 10 articles. In the Citespace co-analysis visualization, cooler colors indicate more distant time, while warmer colors indicate more recent. In the field of ALI/ARDS associated with viral pneumonia, Ashbaugh DG had a greater influence in the early stage, and Peiris JSM had the greatest influence in the middle stage, but there is no co-cited author with high influence recently.

### Co-cited references analysis

**Figure 4:**
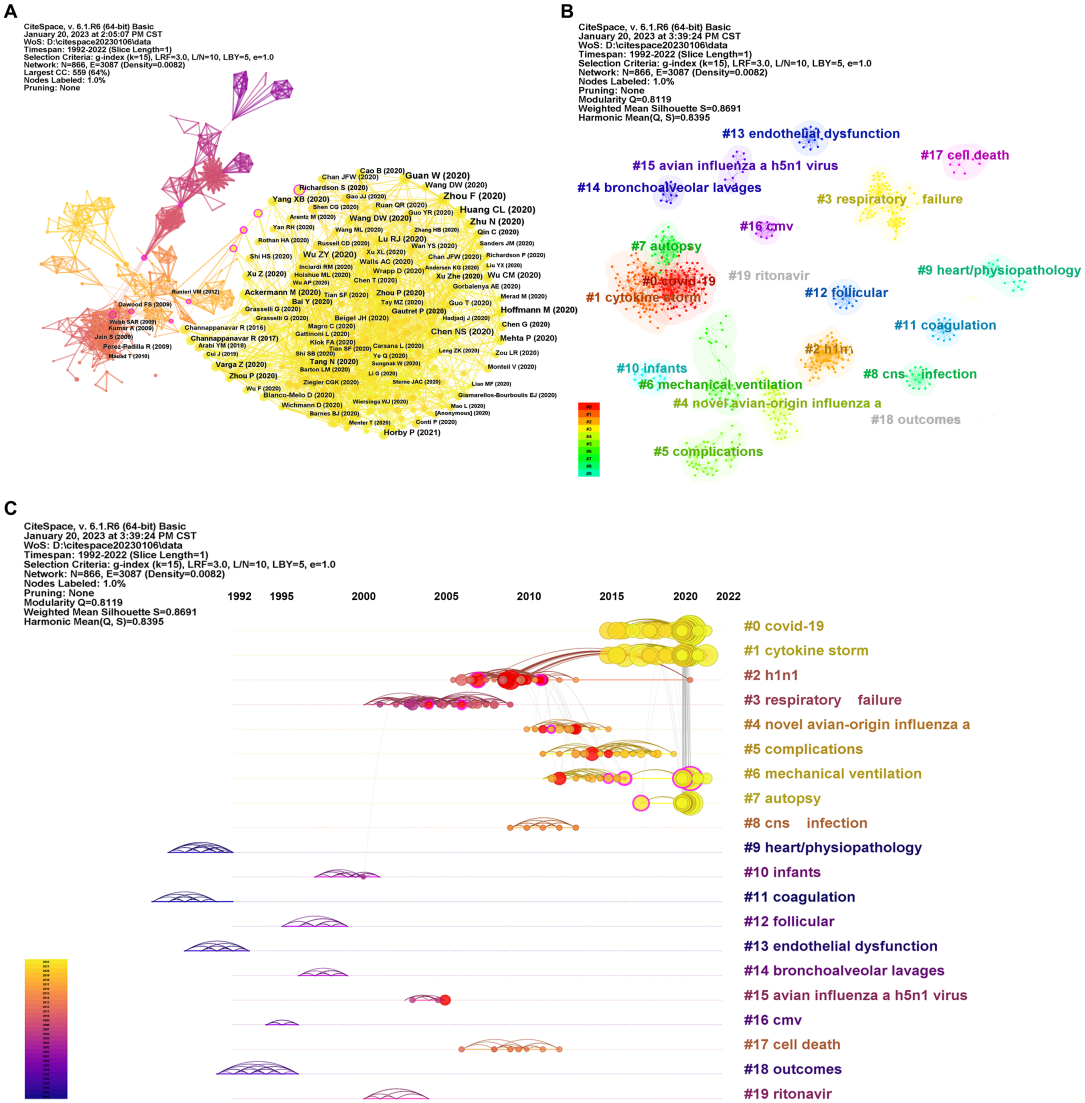
Networks of co-cited references. Each node represents a co-cited references. **(A)** A network diagram of co-cited references. The larger the node, the more frequently it is co-cited. The thicker the connection line, the closer the relationship. The cooler colors indicate more distant time, while warmer colors indicate more recent time. A node with purple ring means high betweenness centrality (>0.1); **(B)** Clusters plots of co-cited references. Different colors represent different clusters of co-cited references; **(C)** Time line and clustering view of co-cited references. The larger the node, the more frequently it is co-cited. The thicker the connection line, the closer the relationship. The cooler colors indicate more distant time, while warmer colors indicate more recent time. A node with purple ring means high betweenness centrality (>0.1), and red ring represents a citation burst.

There are 866 nodes and 3,087 links in co-cited references citation network map as shown in Figure 4A. The top 15 co-cited references in the field of ALI/ARDS associated with viral pneumonia research are displayed in Supplementary Table S1. Among the top 15 most co-cited references, 14 were published in 2020 and 1 was delivered in 2021, all of which were related to COVID-19. ALL papers were in top journals (IF>10): *Lancet* (5), *New England Journal of Medicine* (4), *JAMA-Journal of the American Medical Association* (2), *JAMA Internal Medicine* (1), *Nature* (1), *Lancet Respiratory Medicine* (1), and *Cell* (1). Three references were co-cited over 100 times, and others were co-cited between 45 and 91 times. Among the top 15 co-cited references, no reference had centrality greater than 0.1, three references had relatively high centrality of 0.7 (16), 0.6 (17), and 0.5 (18), and the others had centrality between 0.00 and 0.02.

A total of 113 clusters were calculated, of which 20 clusters were dominant and contained most of the co-cited references (Figure 4B). Details of the most co-cited references in cluster #0 to cluster #19 are available in Supplementary Table S2. In this cluster analysis, the modularity *Q* value of 0.812 was greater than 0.300, and the silhouette value of 0.870 was greater than 0.700, indicating that the clustering results were highly reliable (Figure 4B). Each cluster label was selected by indexing terms from the references based on log-likelihood ratio algorithm. In addition, a timeline map was constructed to display the vital clusters of co-cited references (Figure 4C).

The top 25 co-cited references with the strongest citation bursts are presented in Supplementary Figure S3. The article (19), a review published by Beigel in 2005, was the first reference with the citation bursts appearing in 2006, which was related to human infection with avian influenza A (H5N1). The article (20) published by Peiris in 2004, was the reference with the citation bursts appearing in 2007, which subject matter was related to H5N1 viral pneumonia with respiratory distress. In addition, there were 14 of the top 25 references that showed a citation burst starting in 2010, and all of them were related to H1N1. Two references were mainly related to treatment: the topic of one article published in 2009 by Davies et al. (21) was “Extracorporeal membrane oxygenation for 2009 Influenza A (H1N1) acute respiratory distress syndrome”; the topic of the other article published in 2011 by Martin-Loeches et al. (22) was “Use of early corticosteroid therapy on ICU admission in patients affected by severe pandemic (H1N1)v influenza A infection.” The article written by Perez-Padilla et al. (23) had the strongest burst (17.33), which found novel swine-origin influenza A (H1N1) virus infection could cause acute respiratory distress syndrome, and other articles had a burst strength between 4.66 and 17.33. Mauad et al. (24) summarized the pulmonary pathology of fatal novel human A (H1N1) influenza infection.

### Keywords analysis

**Figure 5:**
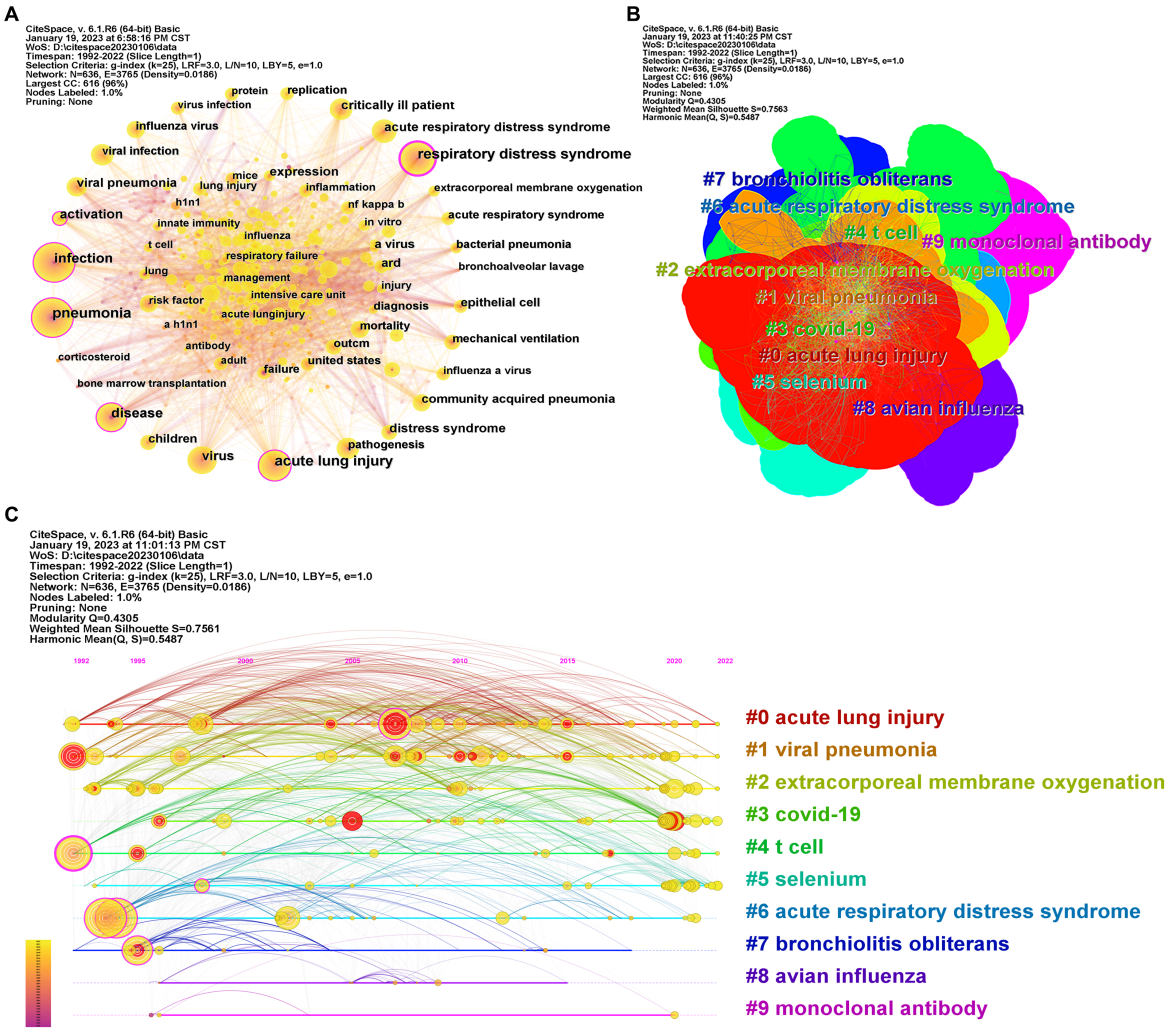
Keywords map. Each node represents a keyword. A co-occurrence relationship between two keywords is represented by links connecting two nodes. **(A)** Network of keywords. The size of each node indicates the frequency of the keywords, whereas the node labels show the keywords. The thicker the connection line, the closer the relationship. The cooler colors indicate more distant time, while warmer colors indicate more recent time. A node with purple ring means high betweenness centrality (>0.1); **(B)** Clusters plots of keywords. Different colors represent different clusters of keywords; **(C)** Time line and clustering view of keywords. The size of each node indicates the frequency of the keywords. The thicker the connection line, the closer the relationship. A node with purple ring means high betweenness centrality (>0.1), and red ring represents a citation burst.

Keywords are the high-level summary. High-frequency and high-centrality keywords often reflect hot research topics in a field (25). A total of 636 keywords were extracted, of which 86 terms appeared more than 10 times, and 10 appeared more than 50 times. The top 15 keywords with the highest frequency and centrality in the field of ALI/ARDS associated with viral pneumonia research are displayed in Table 3. “Pneumonia” ranked first with 169 occurrences, followed by “infection” (133), “acute lung injury” (112), and “respiratory distress syndrome” (108). The rest of the top 15 keywords had frequencies ranging from 41 to 80 occurrences. In addition, “respiratory distress syndrome” (0.24), “acute lung injury” (0.18), “disease” (0.17), “pneumonia” (0.15), “infection” (0.15), and “activation” (0.13) shared “bridge” effects in the keyword co-occurrence map (Figure 5A).

**Table 3:** The top 15 keywords with the highest frequency and centrality in the field of ALI/ARDS associated with viral pneumonia research.

Rank	Keyword	Count	Initial year	Rank	Keyword	Centrality	Initial year
1	Pneumonia	169	1994	1	Respiratory distress syndrome	**0.24**	1992
2	Infection	133	1994	2	Acute lung injury	**0.18**	2007
3	Acute lung injury	112	2007	3	Disease	**0.17**	1995
4	Respiratory distress syndrome	108	1992	4	Pneumonia	**0.15**	1994
5	Coronavirus	80	2005	5	Infection	**0.15**	1994
6	Acute respiratory distress syndrome	68	2002	6	Activation	**0.13**	1998
7	Disease	61	1995	7	Virus	0.09	1992
8	Viral pneumonia	56	1997	8	Epithelial cell	0.08	1998
9	Acute respiratory syndrome	52	2004	9	Acute respiratory distress syndrome	0.07	2002
10	Pathogenesis	50	1998	10	Expression	0.07	1992
11	Viral infection	47	2011	11	A virus	0.07	1994
12	Cytokine storm	43	2020	12	Critically ill patient	0.06	2007
13	Critically ill patient	43	2007	13	Children	0.06	1996
14	Virus	42	1992	14	Viral pneumonia	0.05	1997
15	Mortality	41	1993	15	Distress syndrome	0.05	1995

Bold for high centrality (>0.1).

Clustering knowledge map was built to visualize keyword clusters. Ten clusters were produced by indexing terms from the keywords based on log-likelihood ratio algorithm including #0 acute lung injury #1 viral pneumonia #2 extracorporeal #3 covid-19 #4 t cell #5 selenium #6 acute respiratory distress syndrome #7 bronchlolitis obliterans #8 avian influenza and #9 monoclonal antibody (Figure 5B). The most typical labels in each cluster are shown in Supplementary Table S3. The mean silhouette values of the 10 clusters in the figure was greater than 0.7 indicating good homogeneity suggesting that the analysis results were reliable. Keyword timeline diagram was constructed to explore the time characteristics of the research field reflected by each cluster (Figure 5C)

**Figure 6:**
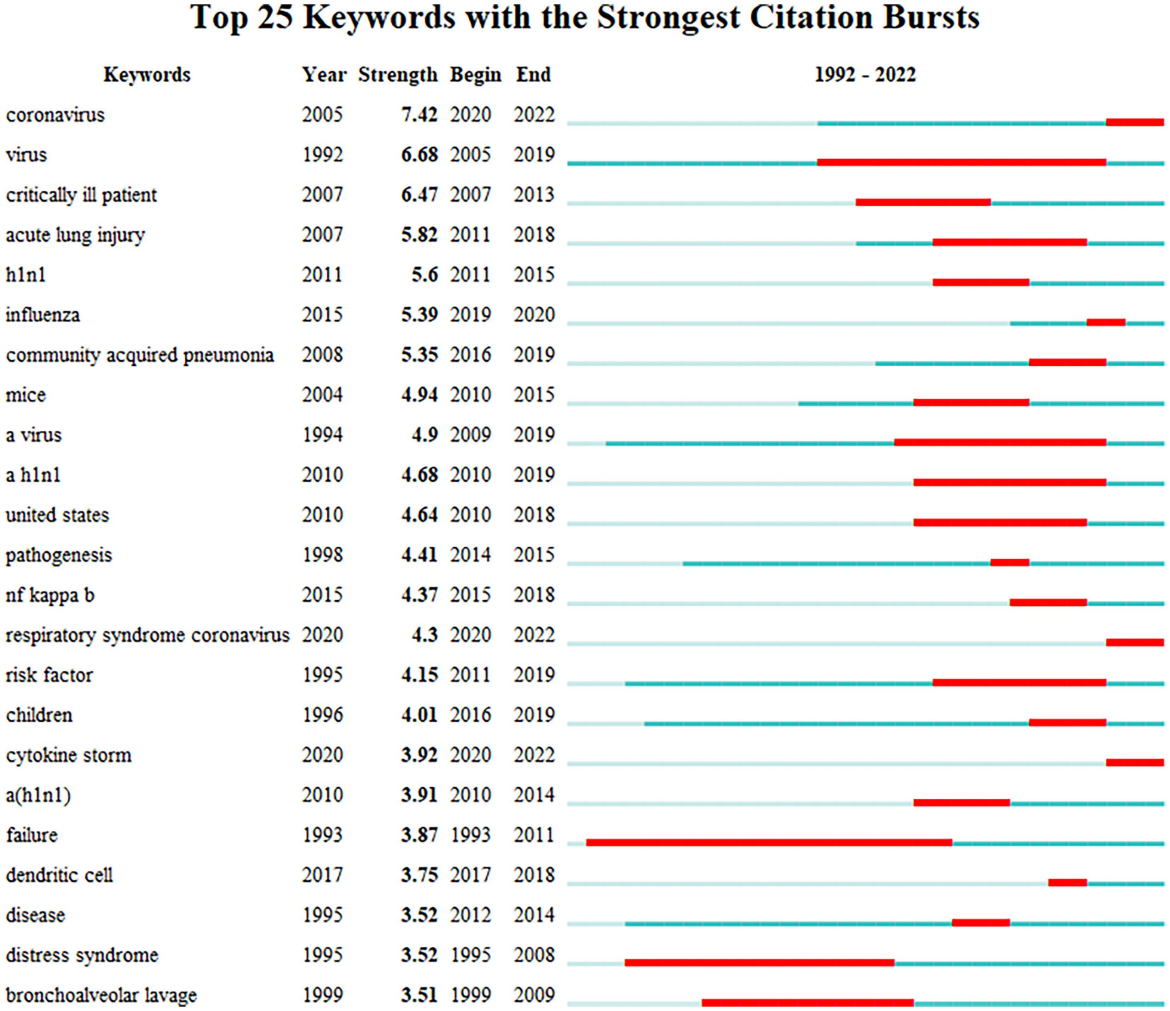
Top 25 keywords with strongest citation bursts. The years between “Begin” and “End” represent the period when the keyword was more influential. Years in light blue mean that the keyword has not yet appeared, years in dark blue mean that the keyword is less influential, and years in red mean that the keyword is more influential.

“Burst keywords” mean that keywords are cited frequently over some time (15). The top 25 keywords with the strongest citation bursts since 1992 are shown in Figure 6, which are also the research frontiers in the field. The red bars indicate the emergence and duration of research hotspots (26). The shortest duration of the burst was 1 years and the longest duration was 18 years. The keywords “failure” (1993–2011), “distress syndrome” (1995–2008), and “virus” (2002–2019) received the longest attention in the past period. Keywords such as “coronavirus” (2020–2022), “respiratory syndrome coronavirus” (2020–2022), and “cytokine storm” (2020–2022) have been used more recently, indicating that these keywords have received enough attention lately and potentially may become future research hotspots. Coronavirus had the strongest citation burst intensity (7.42), followed by virus (6.68), critically ill patient (6.47), acute lung injury (5.82), H1N1 (5.6), influenza (5.39), and other top 25 keywords with a burst strength between 3.51 and 5.35. These words are mainly concentrated in three categories: population of concern for ALI/ARDS associated with viral pneumonia (e.g., critically ill patient and children), causes for ALI/ARDS associated with viral pneumonia (e.g., coronavirus, virus, H1N1, influenza, a virus, a H1N1, and a(H1N1), risk factor, respiratory syndrome coronavirus), pathogenesis of ALI/ARDS associated with viral pneumonia (e.g., pathogenesis, nf kappa b, cytokine storm, and dendritic cell). Combined with the above data and analysis, the major research frontiers in recent years are coronavirus, acute lung injury, cytokine storm, and respiratory syndrome.

## Discussion

This study analyzed the number of countries, institutions, co-cited journals, and authors, etc. in the research on ALI/ARDS associated with viral pneumonia. The results show that over the past 31 years, there has been a gradual increase in research on this topic worldwide, with an explosive growth in 2020, which was significantly associated with the outbreak of novel coronavirus in 2019. Through the visualization map, the communication and contact among countries, institutions and authors can be seen intuitively (Figure 2; Supplementary Figures S1, S2). It is not difficult to find that the cooperation between countries is closer than that between institutions, and the cooperation between institutions is also significantly closer than that between authors. Countries/Regions with centrality greater than 0.1, occupied an important position in the research of ALI/ARDS associated with viral pneumonia. However, despite the relatively small number of publications in Canada, England, Spain, and Saudi Arabia, the impact was significant. The United States and China were in a leading position in the number of publications in this field, but their influence still needed to be strengthened, with the centrality of 0.08 and 0.02, respectively. In addition, the top 15 institutions are all universities, indicating that institutions of higher learning are the mainstay of this field and play an important role in research on this topic (Table 1).

Co-cited references not only present the core literature that plays a pivotal role in the evolutionary process within a field, but also unmask the changing trends in research priorities (27). Through the timeline map of co-cited references, it can be found that in terms of time distribution, clustering was relatively concentrated, mainly including #0 covid-19, #1 cytokine storm, #2 H1N1, #3 respiratory failure, #4 novel avian-origin influenza, #5 complications, #6 mechanical ventilation, and #7 autopsy. It was obvious that #0 covid-19, #1 cytokine storm, #6 mechanical ventilation, and #7 autopsy clusters mainly occurred from 2015 to 2022, indicating that these clusters are hot spots for ALI/ARDS associated with viral pneumonia research (Figure 4C). It is worth noting that the top 15 most co-cited references were all related to COVID-19, which is clearly relevant to the ongoing COVID-19 outbreak since 2019. In addition, the top 25 co-cited references with the strongest outbreak were mainly related to H1N1, involving the pathological mechanism (23) and treatment (22) of ALI/ARDS caused by H1N1, which was related to the pandemic of 2009 H1N1. Martin-Loeches et al. (22) showed that early use of corticosteroids in patients with ALI/ARDS due to pandemic influenza A H1N1 infection did not lead to better outcomes and was associated with an increased risk of superinfection. This has reference value for the current exploration of the treatment of ALI/ARDS caused by COVID-19. In addition, Davies et al. (21) found Extracorporeal Membrane Oxygenation as a salvage treatment modality for ALI/ARDS associated with viral pneumonia.

In order to explore the research trends and the latest hot spots, a cluster analysis was performed on the keywords, and a total of 10 clusters were obtained. Different from other viruses, SARS in 2002, H5N1 in 2003, H1N1 in 2009, MERS-CoV in 2012 and COVID-19 in 2019 are more likely to cause ALI/ARDS, which in turn cause greater harm to human public health. This may partly explain why they have become the focus of research in the field. Cytokine storm is considered to be one of the main causes of ALI/ARDS associated with viral pneumonia (28). Studies have shown that delayed release of IFN in the early stage of MERS-CoV infection can hinder the body’s antiviral response (29). Later, the rapidly increasing cytokines and chemokines attract many inflammatory cells, such as neutrophils and monocytes, leading to excessive infiltration of inflammatory cells into lung tissue, which causes ALI/ARDS (29). The treatment of ALI/ARDS associated with viral pneumonia mainly includes two aspects: mechanical ventilation therapy such as extracorporeal membrane oxygenation therapy and non-mechanical ventilation therapy such as alveolar surfactant supplement therapy and moderate use of glucocorticoids. Sahebnasagh et al. (30) suggest that a neutrophil elastase inhibitor (sivelestat) may be a promising treatment option for patients with acute lung injury/ARDS in COVID-19, but this view needs to be tested in large clinical trials. In addition, studies have shown that PTP1B inhibitors protect against acute lung injury and regulate CXCR4 signaling in neutrophils, which means that the use of PTB1B inhibitors in patients with severe viral pneumonia may have a preventive effect on ALI/ARDS (31).

Compared with previous research in this field, this study has some strengths. Most importantly, this is the first bibliometric study of ALI/ARDS associated with viral pneumonia. In addition, the online bibliometric analysis platformand and bibliometric software (Citespace) were used together for analysis and visualization, which increased the richness of the results. Although the analysis and visualization in this study are relatively comprehensive and objective, it has several limitations. First, the search date for included publications in our study was up to January 6, 2023, but new articles were continuously published that were not included in our study. Second, our study only included publications in English, and contributions from publications in other languages may have been overlooked. Finally, due to the format requirements of Citespace, we ended up counting only publications in the WOSCC database, which may have missed articles from other databases, such as Pubmed, Medline, and Scopus. However, because of the significant cross-replication of the literature in the various databases and the authority of the WoSCC database, we consider that this work still can be applied to present the overall situation and general trend for this field.

## Conclusion

Bibliometric analysis demonstrates, in the field of ALI/ARDS associated with viral pneumonia, the United States and China have been the leader. The United States ranked first with 320 publications, followed by China with 158 papers. In addition, Canada is the country with the highest influence in this field. Fudan University, Northwestern University and Capital Medical University were the most important institutions. The *New England Journal of Medicine* is the most frequently co-cited journal, but none of the co-cited journals in this field had a betweenness centrality greater than 0.1, indicating that the influence of co-cited journals needs to be improved. Bem RA is the most highly published author in this field, and David G Ashbaugh is the most influential co-cited author. With the development of research, the study focus has gradually shifted from pathological mechanism analysis to the identification of high-risk groups, treatment and management, prevention and risk factor analysis. At the same time, the continuous outbreak of keywords such as “coronavirus,” “respiratory syndrome coronavirus” and “cytokine storm” indicates that these keywords will continue to be research hotspots in the future. In summary, our study objectively and comprehensively shows the research trends, hotspots, frontiers, existing problems and deficiencies in the field of ALI/ARDS associated with viral pneumonia through bibliometric analysis, hoping to help scholars in this field to quickly identify their strengths and weaknesses, so as to enrich and improve the development of this field.

## Data availability statement

The original contributions presented in the study are included in the article/Supplementary material, further inquiries can be directed to the corresponding authors.

## Author contributions

BZ and ZZ were involved in the conception and study design. LZ and SM were responsible for document retrieval. LZ and BZ were involved in the writing and revision of the manuscript. LZ was responsible for the data analysis. All authors contributed to the article and approved the submitted version.

## Conflict of interest

The authors declare that the research was conducted in the absence of any commercial or financial relationships that could be construed as a potential conflict of interest.

## Publisher’s note

All claims expressed in this article are solely those of the authors and do not necessarily represent those of their affiliated organizations, or those of the publisher, the editors and the reviewers. Any product that may be evaluated in this article, or claim that may be made by its manufacturer, is not guaranteed or endorsed by the publisher.

## Supplementary material

The Supplementary material for this article can be found online at: https://www.frontiersin.org/articles/10.3389/fmed.2023.1158519/full#supplementary-material

## Footnotes

^1^ http://bibliometric.com/

## References

[1] Liu C Xiao K Xie LX. Advances in the use of exosomes for the treatment of ALI/ARDS. Front Immunol. (2022) 13:13. doi: 10.3389/fimmu.2022.971189, PMID: 36016948 PMC9396740

[2] Kaku S Nguyen CD Htet NN Tutera D Barr J Paintal HS. Acute respiratory distress syndrome: etiology, pathogenesis, and summary on management. J Intensive Care Med. (2020) 35:723–37. doi: 10.1177/0885066619855021 31208266

[3] Figueiredo LT. Viral pneumonia: epidemiological, clinical, pathophysiological and therapeutic aspects. Jornal Brasileiro de Pneumologia. (2009) 35:899–906. doi: 10.1590/s1806-37132009000900012, PMID: 19820817

[4] Pagliano P Sellitto C Conti V Ascione T Esposito S. Characteristics of viral pneumonia in the COVID-19 era: an update. Infection. (2021) 49:607–16. doi: 10.1007/s15010-021-01603-y, PMID: 33782861 PMC8006879

[5] Ruuskanen O Lahti E Jennings LC Murdoch DR. Viral pneumonia. Lancet. (2011) 377:1264–75. doi: 10.1016/S0140-6736(10)61459-6, PMID: 21435708 PMC7138033

[6] Wu L Wu H Ou T Huang H Duan L Li W. Mapping theme trends and recognizing hot spots in viral pneumonia: a bibliometric analysis of global research. Am J Transl Res. (2022) 14:2972–87. PMID: 35702075 PMC9185022

[7] Zhong NS Zheng BJ Li YM Poon LLM Xie ZH Chan KH. Epidemiology and cause of severe acute respiratory syndrome (SARS) in Guangdong, People's Republic of China, in February, 2003. Lancet. (2003) 362:1353–8. doi: 10.1016/s0140-6736(03)14630-2, PMID: 14585636 PMC7112415

[8] Baharoon S Memish ZA. MERS-CoV as an emerging respiratory illness: a review of prevention methods. Travel Med Infect Dis. (2019) 32:101520. doi: 10.1016/j.tmaid.2019.101520, PMID: 31730910 PMC7110694

[9] Perlman S. Another decade, another coronavirus. N Engl J Med. (2020) 382:760–2. doi: 10.1056/NEJMe2001126, PMID: 31978944 PMC7121143

[10] Tran B Pham T Ha G Ngo A Nguyen L Vu T. A bibliometric analysis of the global Research Trend in child maltreatment. Int J Environ Res Public Health. (2018) 15:1456. doi: 10.3390/ijerph15071456, PMID: 29996540 PMC6069266

[11] Wei N Xu Y Li Y Shi J Zhang X. A bibliometric analysis of T cell and atherosclerosis. Front Immunol. (2022) 13:948314. doi: 10.3389/fimmu.2022.948314, PMID: 36311729 PMC9606647

[12] YildirimFGulhanPYKaramanIKurutkanMN. Bibliometric analysis of acute respiratory distress syndrome (ARDS) studies published between 1980 and 2020. Adv Clin Exp Med (2022) 31(7):807–813. doi: 10.17219/acem/15055535699587

[13] Jiang R Cao M Mei S Guo S Zhang W Ji N. Trends in metabolic signaling pathways of tumor drug resistance: a scientometric analysis. Front. Oncologia. (2022) 12:981406. doi: 10.3389/fonc.2022.981406 PMC9641273 36387132

[14] Dang Q Luo Z Ouyang C Wang L. First systematic review on health communication using the CiteSpace software in China: exploring its research hotspots and Frontiers. Int J Environ Res Public Health. (2021) 18:13008. doi: 10.3390/ijerph182413008, PMID: 34948617 PMC8702194

[15] Luo H Cai Z Huang Y Song J Ma Q Yang X. Study on pain catastrophizing from 2010 to 2020: a bibliometric analysis via CiteSpace. Front Psychol. (2021) 12:759347. doi: 10.3389/fpsyg.2021.759347, PMID: 34975649 PMC8718514

[16] Yang XB Yu Y Xu JQ Shu HQ Xia JA Liu H. Clinical course and outcomes of critically ill patients with SARS-CoV-2 pneumonia in Wuhan, China: a single-centered, retrospective, observational study. Respir Med. (2020) 8:475–81. doi: 10.1016/s2213-2600(20)30079-5, PMID: 32105632 PMC7102538

[17] Zhou F Yu T du R Fan GH Liu Y Liu ZB. Clinical course and risk factors for mortality of adult inpatients with COVID-19 in Wuhan, China: a retrospective cohort study. Lancet. (2020) 395:1054–62. doi: 10.1016/s0140-6736(20)30566-3, PMID: 32171076 PMC7270627

[18] Lu RJ Zhao X Li J Niu PH Yang B Wu HL. Genomic characterisation and epidemiology of 2019 novel coronavirus: implications for virus origins and receptor binding. Lancet. (2020) 395:565–74. doi: 10.1016/s0140-6736(20)30251-8, PMID: 32007145 PMC7159086

[19] Beigel H Farrar H Han AM Hayden FG Hyer R de Jong MD. Avian influenza a (H5N1) infection in humans. N Engl J Med. (2005) 353:1374–85. doi: 10.1056/NEJMra052211 16192482

[20] Peiris JSM Yu WC Leung CW Cheung CY Ng WF Nicholls JM. Re-emergence of fatal human influenza a subtype H5N1 disease. Lancet. (2004) 363:617–9. doi: 10.1016/s0140-6736(04)15595-5, PMID: 14987888 PMC7112424

[21] Davies A Jones D Bailey M Beca J Bellomo R Blackwell N. Extracorporeal membrane oxygenation for 2009 influenza a(H1N1) acute respiratory distress syndrome. JAMA. (2009) 302:1888–95. doi: 10.1001/jama.2009.1535 19822628

[22] The ESICM H1N1 Registry Contributors Martin-Loeches I Lisboa T Rhodes A Moreno RP Silva E. Use of early corticosteroid therapy on ICU admission in patients affected by severe pandemic (H1N1)v influenza a infection. Intensive Care Med. (2011) 37:272–83. doi: 10.1007/s00134-010-2078-z, PMID: 21107529 PMC7079858

[23] Perez-Padilla R de la Rosa-Zamboni D Ponce de Leon S Hernandez M Quiñones-Falconi F Bautista E. Pneumonia and respiratory failure from swine-origin influenza a (H1N1) in Mexico. N Engl J Med. (2009) 361:680–9. doi: 10.1056/NEJMoa0904252, PMID: 19564631

[24] Mauad T Hajjar LA Callegari GD da Silva LFF Schout D Galas F. Lung pathology in fatal novel human influenza a (H1N1) infection. Am J Respir Crit Care Med. (2010) 181:72–9. doi: 10.1164/rccm.200909-1420OC, PMID: Retraction in: Am J Respir Crit Care Med. 2011 Nov 1;184(9):1086; author reply 1086 19875682

[25] Zhong D Li Y Huang Y Hong X Li J Jin R. Molecular mechanisms of exercise on Cancer: a Bibliometrics study and visualization analysis via CiteSpace. Front Mol Biosci. (2021) 8:797902. doi: 10.3389/fmolb.2021.797902, PMID: 35096970 PMC8794585

[26] Chen B Fu Y Song G Zhong W Guo J. Research trends and hotspots of exercise for Alzheimer's disease: a bibliometric analysis. Front Aging Neurosci. (2022) 14:984705. doi: 10.3389/fnagi.2022.984705 36158544 PMC9490271

[27] Tang C Liu D Fan Y Yu J Li C Su J. Visualization and bibliometric analysis of cAMP signaling system research trends and hotspots in cancer. J Cancer. (2021) 12:358–70. doi: 10.7150/jca.47158, PMID: 33391432 PMC7738981

[28] Ye Q Wang BL Mao JH. The pathogenesis and treatment of the 'Cytokine Storm' in COVID-19. J Infect. (2020) 80:607–13. doi: 10.1016/j.jinf.2020.03.037, PMID: 32283152 PMC7194613

[29] Channappanavar R Fehr AR Zheng J Wohlford-Lenane C Abrahante JE Mack M. IFN-I response timing relative to virus replication determines MERS coronavirus infection outcomes. J Clin Investig. (2019) 129:3625–39. doi: 10.1172/jci126363, PMID: 31355779 PMC6715373

[30] Sahebnasagh A Saghafi F Safdari M Khataminia M Sadremomtaz A Talaei Z. Neutrophil elastase inhibitor (sivelestat) may be a promising therapeutic option for management of acute lung injury/acute respiratory distress syndrome or disseminated intravascular coagulation in COVID-19. J Clin Pharm Ther. (2020) 45:1515–9. doi: 10.1111/jcpt.13251 32860252

[31] Song DY Adrover JM Felice C Christensen LN He XY Merrill JR. PTP1B inhibitors protect against acute lung injury and regulate CXCR4 signaling in neutrophils. Jci. Insight. (2022) 7:e158199. doi: 10.1172/jci.insight.158199 PMC9431713 35866483

