# Supplementary material for: Trends in research on acute lung injury/acute respiratory distress syndrome associated with viral pneumonia from 1992 to 2022: a 31-year bibliometric analysis
Source: Front Med (Lausanne). 2023 Jun 9;10:1158519. doi: 10.3389/fmed.2023.1158519 (PMC10288490; doi:10.3389/fmed.2023.1158519)
Supplement: Supplementary file 1 [file Data_Sheet_1.pdf]

Supplementary material

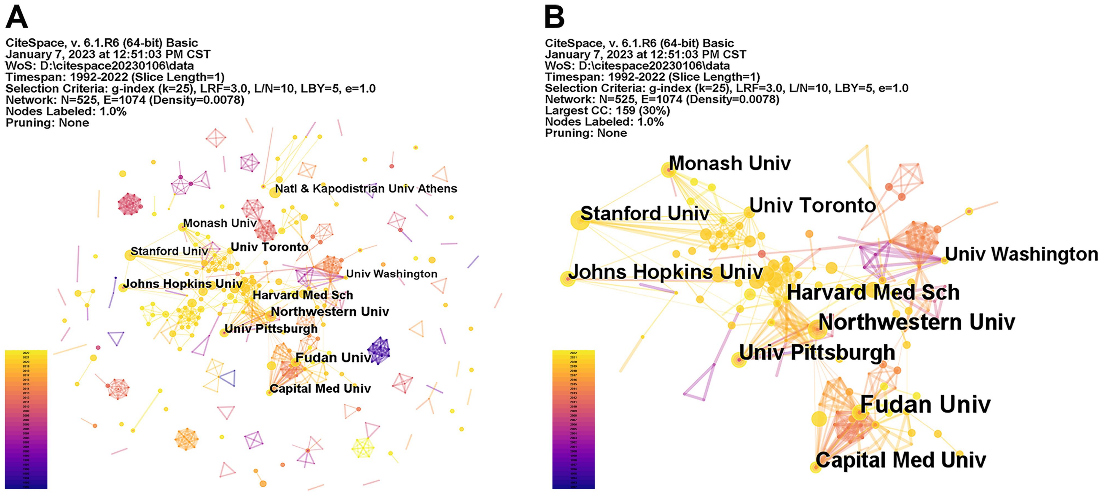

Figure S1. **(A)** Collaborative network of institutions; **(B)** The largest network of institutions cooperation. Each node represents an institution. The bigger the node, the more articles the institution published. The thicker the connection line, the closer the relationship. The cooler colors indicate more distant time, while warmer colors indicate more recent time.

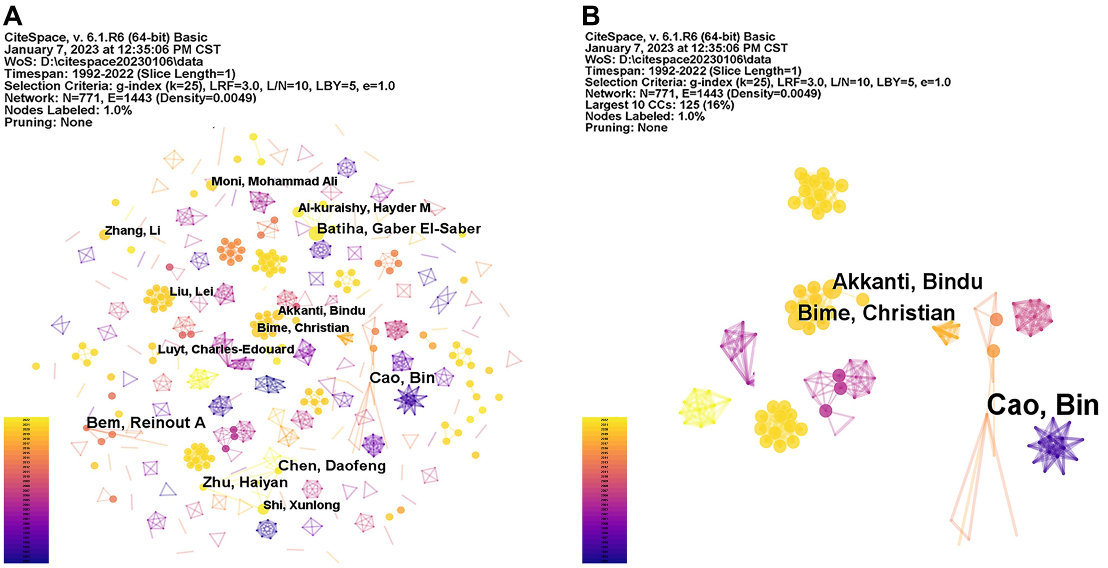

Figure S2. **(A)** Collaborative network of authors; **(B)** The largest network of authors cooperation. Each node represents an author. The bigger the node, the more articles the institution published. The thicker the connection line, the closer the relationship. The cooler colors indicate more distant time, while warmer colors indicate more recent time.

Top 25 References with the Strongest Citation Bursts

References	Year	Strength	Begin	End	1992 - 2022
Perez-Padilla R, 2009, NEW ENGL J MED, V361, P680, DOI 10.1056/NEJMoa0904252, DOI	2009	**17.33**	2010	2013	
Jain S, 2009, NEW ENGL J MED, V361, P1935, DOI 10.1056/NEJMoa0906695, DOI	2009	**11.73**	2010	2013	
Kumar A, 2009, JAMA-J AM MED ASSOC, V302, P1872, DOI 10.1001/jama.2009.1496, DOI	2009	**11.11**	2010	2013	
Dawood FS, 2009, NEW ENGL J MED, V360, P2605, DOI 10.1056/NEJMoa0903810, DOI	2009	**10.28**	2010	2014	
Ranieri VM, 2012, JAMA-J AM MED ASSOC, V307, P2526, DOI 10.1001/jama.2012.5669, DOI	2012	**10.1**	2013	2017	
Mauad T, 2010, AM J RESP CRIT CARE, V181, P72, DOI 10.1164/rccm.200909-1420OC, DOI	2010	**9.87**	2010	2013	
Webb SAR, 2009, NEW ENGL J MED, V361, P1925, DOI 10.1056/NEJMoa0908481, DOI	2009	**9.45**	2010	2012	
Dominguez-Cherit G, 2009, JAMA-J AM MED ASSOC, V302, P1880, DOI 10.1001/jama.2009.1536, DOI	2009	**8.63**	2010	2013	
Short KR, 2014, LANCET INFECT DIS, V14, P57, DOI 10.1016/S1473-3099(13)70286-X, DOI	2014	**7.77**	2016	2019	
Jamieson DJ, 2009, LANCET, V374, P451, DOI 10.1016/S0140-6736(09)61304-0, DOI	2009	**7.4**	2010	2013	
Itoh Y, 2009, NATURE, V460, P1021, DOI 10.1038/nature08260, DOI	2009	**7.4**	2010	2013	
Gao HN, 2013, NEW ENGL J MED, V368, P2277, DOI 10.1056/NEJMoa1305584, DOI	2013	**7.03**	2014	2018	
Louie JK, 2009, JAMA-J AM MED ASSOC, V302, P1896, DOI 10.1001/jama.2009.1583, DOI	2009	**6.78**	2010	2013	
Chowell G, 2009, NEW ENGL J MED, V361, P674, DOI 10.1056/NEJMoa0904023, DOI	2009	**6.78**	2010	2013	
Mandell LA, 2007, CLIN INFECT DIS, V44, PS27, DOI 10.1086/511159, DOI	2007	**6.65**	2008	2012	
Davies A, 2009, JAMA-J AM MED ASSOC, V302, P1888, DOI 10.1001/jama.2009.1535, DOI	2009	**6.64**	2010	2014	
Rello J, 2009, CRIT CARE, V13, P0, DOI 10.1186/cc8044, DOI	2009	**6.29**	2010	2012	
Chen Y, 2013, LANCET, V381, P1916, DOI 10.1016/S0140-6736(13)60903-4, DOI	2013	**5.79**	2014	2017	
Gao RB, 2013, NEW ENGL J MED, V368, P1888, DOI 10.1056/NEJMoa1304459, DOI	2013	**5.63**	2013	2018	
Beigel H, 2005, NEW ENGL J MED, V353, P1374, DOI 10.1056/NEJMra052211, DOI	2005	**5.29**	2006	2009	
Napolitano L. M., 2009, MORBIDITY AND MORTALITY WEEKLY REPORT, V58, P749	2009	**5.2**	2010	2011	
de Jong MD, 2006, NAT MED, V12, P1203, DOI 10.1038/nm1477, DOI	2006	**5.17**	2008	2010	
Bautista E, 2010, NEW ENGL J MED, V362, P1708, DOI 10.1056/NEJMra1000449, DOI	2010	**5.12**	2011	2013	
Martin-Loeches I, 2011, INTENS CARE MED, V37, P272, DOI 10.1007/s00134-010-2078-z, DOI	2011	**4.99**	2012	2016	
Peiris JSM, 2004, LANCET, V363, P617, DOI 10.1016/S0140-6736(04)15595-5, DOI	2004	**4.66**	2007	2009	

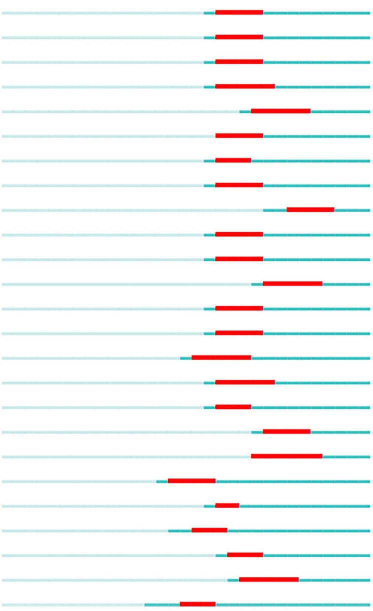

Figure S3. Top 25 references with strongest citation bursts. The timeline is depicted as a dark blue line, and the time interval that a subject was found to have a burst is shown as a red segment which indicated the beginning year, the ending year, and the duration of the burst. Years in light blue mean that the keyword has not yet appeared.

Table S1. The top 15 co-cited references in the field of ALI/ARDS associated with viral pneumonia research.

Rank	Title	Total citations	Centrality	Journal[a]	First author	Year
1	Clinical features of patients infected with 2019 novel coronavirus in Wuhan, China	145	0.01	*Lancet*	Huang CL	2020
2	Clinical course and risk factors for mortality of adult inpatients with COVID-19 in Wuhan, China: a retrospective cohort study	135	0.06	*Lancet*	Zhou F	2020
3	Clinical characteristics of coronavirus disease 2019 in China	108	0.01	*New Engl J Med*	Guan W	2020
4	A novel coronavirus from patients with pneumonia in China, 2019	91	0.02	*New Engl J Med*	Zhu N	2020
5	SARS-CoV-2 cell entry depends on ACE2 and TMPRSS2 and is blocked by a clinically proven protease inhibitor	84	0.01	*Cell*	Hoffmann M	2020
6	Epidemiological and clinical characteristics of 99 cases of 2019 novel coronavirus pneumonia in Wuhan, China: a descriptive study	73	0.02	*Lancet*	Chen NS	2020
7	Characteristics of and important lessons from the coronavirus disease 2019 (COVID-19) outbreak in China summary of a report of 72,314 cases from the Chinese Center for Disease Control and Prevention	67	0.00	*Jama-J Am Med Assoc*	Wu ZY	2020
8	Risk factors associated with acute respiratory distress syndrome and death in patients with coronavirus disease 2019 pneumonia in Wuhan, China	62	0.01	*Jama Intern Med*	Wu CM	2020
9	Genomic characterisation and epidemiology of 2019 novel coronavirus: implications for virus origins and receptor binding	58	0.05	*Lancet*	Lu RJ	2020
10	COVID-19: consider cytokine storm syndromes and immunosuppression	56	0.01	*Lancet*	Mehta P	2020
11	Dexamethasone in hospitalized patients with Covid-19	56	0.00	*New Engl J Med*	Horby P	2021
12	Clinical characteristics of 138 hospitalized patients with 2019 novel coronavirus-infected pneumonia in Wuhan, China	54	0.00	*Jama-J Am Med Assoc*	Wang DW	2020
13	Pulmonary vascular endothelialitis, thrombosis, and angiogenesis in Covid-19	52	0.00	*New Engl J Med*	Ackermann M	2020
14	Clinical course and outcomes of critically ill patients with SARS-CoV-2 pneumonia in Wuhan, China: a single-centered, retrospective, observational study	49	0.07	*Lancet Resp Med*	Yang XB	2020
15	A pneumonia outbreak associated with a new coronavirus of probable bat origin	45	0.01	*Nature*	Zhou P	2020

[a]Journal names according to Index of Medical Journal Abbreviations.

Table S2. Details of the most co-cited references in co-cited references cluster #0 to cluster #19.

Cluster	Author	Year	Journal[a]	Count	Centrality	DOI
#0	Huang CL	2020	*LANCET*	145	0.01	10.1016/S0140-6736(20)30183-5
#1	Wu CM	2020	*JAMA INTERN MED*	62	0.01	10.1001/jamainternmed.2020.0994
#2	Perez-Padilla R	2009	*NEW ENGL J MED*	28	0.01	10.1056/NEJMoa0904252
#3	de Jong MD	2006	*NAT MED*	8	**0.21**	10.1038/nm1477
#4	Gao HN	2013	*NEW ENGL J MED*	11	0.03	10.1056/NEJMoa1305584
#5	Short KR	2014	*LANCET INFECT DIS*	12	0.07	10.1016/S1473-3099(13)70286-X
#6	Richardson S	2020	*JAMA-J AM MED ASSOC*	34	**0.12**	10.1001/jama.2020.6775
#7	Ackermann M	2020	*NEW ENGL J MED*	52	0.00	10.1056/NEJMoa2015432
#8	Desforges M	2013	*J VIROL*	2	0.00	10.1128/JVI.02699-12
#9	Marini JJ	1992	*AM REV RESPIR DIS*	2	0.00	10.1164/ajrccm/146.1.2
#10	Ware LB	2000	*NEW ENGL J MED*	4	0.05	10.1056/NEJM200005043421806
#11	Emilie D	1988	*EUR J IMMUNOL*	1	0.00	10.1002/eji.1830181226
#12	Mcdonough KA	1995	*INFECT IMMUN*	1	0.00	10.1128/IAI.63.12.4802-4811.1995
#13	Martin K	1991	*CELL*	1	0.00	10.1016/0092-8674(91)90576-K
#14	Papadopoulos NG	1999	*J MED VIROL*	1	0.00	10.1002/(SICI)1096-9071(199905)58:1<100::AID-JMV16>3.0.CO;2-D
#15	Beigel H	2005	*NEW ENGL J MED*	8	0.00	10.1056/NEJMra052211
#16	Bando K	1995	*J THORAC CARDIOV SUR*	1	0.00	10.1016/S0022-5223(05)80003-0
#17	Nair H	2010	*LANCET*	4	0.00	10.1016/S0140-6736(10)60206-1
#18	Barton BE	1993	*INFECT IMMUN*	1	0.00	10.1128/IAI.61.4.1496-1499.1993
#19	Bridges CB	2000	*J INFECT DIS*	1	0.00	10.1086/315213

[a]Journal names according to Index of Medical Journal Abbreviations. Bold for high centrality (> 0.1).

Table S3. Details of each cluster of keywords.

Cluster	Label	Size	Silhouette	Mean (year)
#0	acute lung injury; expression; mice; protein; respiratory syncytial virus	106	0.781	2007
#1	viral pneumonia; viral infection; critical care; public health; community-acquired pneumonia	91	0.722	2010
#2	extracorporeal membrane oxygenation; mechanical ventilation; respiratory failure; antiviral therapy; adult respiratory distress syndrome	81	0.709	2006
#3	covid-19; therapy; coronavirus; viral pneumonia; china	80	0.612	2014
#4	t cell; foal; tissue injury; horse; covid-19	65	0.759	2010
#5	selenium; vitamin d; oxidative stress; traditional chinese medicine; endothelial cells	59	0.799	2012
#6	acute respiratory distress syndrome; infection; coagulopathy; chest radiograph; pneumonia	54	0.752	2008
#7	bronchiolitis obliterans; bone marrow transplantation; disease; cytokine storms; intra thoracic manifestation	47	0.925	2003
#8	avian influenza; risk; maternal influenza; herpes simplex; influenza in birds	23	0.901	2005
#9	monoclonal antibody; interferon gamma; interleukin-6;bacterial superantigen; idiopathic pulmonary fibrosis	10	0.976	2000
